# Decomposed Temporal Complexity Analysis of Neural Oscillations and Machine Learning Applied to Alzheimer’s Disease Diagnosis

**DOI:** 10.3389/fpsyt.2020.531801

**Published:** 2020-09-03

**Authors:** Naoki Furutani, Yuta Nariya, Tetsuya Takahashi, Sarah Noto, Albert C. Yang, Tetsu Hirosawa, Masafumi Kameya, Yoshio Minabe, Mitsuru Kikuchi

**Affiliations:** ^1^Department of Psychiatry and Neurobiology, Graduate School of Medical Science, Kanazawa University, Kanazawa, Japan; ^2^Faculty of Medicine, The University of Tokyo, Tokyo, Japan; ^3^Research Center for Child Mental Development, Kanazawa University, Kanazawa, Japan; ^4^Faculty of Nursing, National College of Nursing, Tokyo, Japan; ^5^Division of Interdisciplinary Medicine and Biotechnology, Beth Israel Deaconess Medical Center/Harvard Medical School, Boston, MA, United States; ^6^Institute of Brain Science, National Yang-Ming University, Taipei, Taiwan

**Keywords:** alpha oscillation, Alzheimer’s disease (AD), amplitude complexity, ensemble empirical mode decomposition (EEMD), magnetoencephalography (MEG), multiscale entropy (MSE), phase complexity, sparse autoencoder (SAE)

## Abstract

Despite growing evidence of aberrant neuronal complexity in Alzheimer’s disease (AD), it remains unclear how this variation arises. Neural oscillations reportedly comprise different functions depending on their own properties. Therefore, in this study, we investigated details of the complexity of neural oscillations by decomposing the oscillations into frequency, amplitude, and phase for AD patients. We applied resting-state magnetoencephalography (MEG) to 17 AD patients and 21 healthy control subjects. We first decomposed the source time series of the MEG signal into five intrinsic mode functions using ensemble empirical mode decomposition. We then analyzed the temporal complexities of these time series using multiscale entropy. Results demonstrated that AD patients had lower complexity on short time scales and higher complexity on long time scales in the alpha band in temporal regions of the brain. We evaluated the alpha band complexity further by decomposing it into amplitude and phase using Hilbert spectral analysis. Consequently, we found lower amplitude complexity and higher phase complexity in AD patients. Correlation analyses between spectral complexity and decomposed complexities revealed scale-dependency. Specifically, amplitude complexity was positively correlated with spectral complexity on short time scales, whereas phase complexity was positively correlated with spectral complexity on long time scales. Regarding the relevance of cognitive function to the complexity measures, the phase complexity on the long time scale was found to be correlated significantly with the Mini-Mental State Examination score. Additionally, we examined the diagnostic utility of the complexity characteristics using machine learning (ML) methods. We prepared a feature pool using multiple sparse autoencoders (SAEs), chose some discriminating features, and applied them to a support vector machine (SVM). Compared to the simple SVM and the SVM after feature selection (FS + SVM), the SVM with multiple SAEs (SAE + FS + SVM) had improved diagnostic accuracy. Through this study, we 1) advanced the understanding of neuronal complexity in AD patients using decomposed temporal complexity analysis and 2) demonstrated the effectiveness of combining ML methods with information about signal complexity for the diagnosis of AD.

## Introduction

Alzheimer’s disease (AD), the most common type of dementia, presents clinical symptoms such as memory loss, word-finding difficulties, and visual/spatial problems. The neuropathological features of AD include neuronal loss, neurofibrillary entanglement, and senile plaques, which progress insidiously for decades before the onset of readily apparent symptoms; then they spread widely throughout the brain (1–3). These histopathological changes are thought to be observed as disruptions of local neural connections in early AD (4, 5). Gradually, the changes proceed to impair the long-distance brain network (6, 7). Although numerous efforts have been directed at developing objective diagnostic methods, AD diagnosis is still based mainly on clinical symptoms.

A possible biomarker for use in AD diagnosis is alteration of oscillatory brain activity. The most widely known oscillatory changes in AD patients are spectral changes, which include slowing of the peak frequency and increase in slow oscillations (8), even in the preclinical phase (9, 10). The recent advent of the use of complex network analysis in electroencephalography (EEG) and magnetoencephalography (MEG) studies has revealed abnormal neural oscillations of AD (8, 11, 12). One possible diagnostic measure of AD is the temporal complexity of neural oscillations. Particularly, sample entropy (SampEn) and multiscale entropy (MSE), a measure of SampEn at various time scales, have been well studied (13–16). Fundamentally, MSE is computed for broadband oscillations because the neural complexity of each time scale corresponds to their relevant frequency range (15). However, oscillatory components of each frequency range respectively reflect different neural functions (17, 18). Additionally, it is difficult to ignore the possibility that each neural function (i.e., each oscillatory component) is influenced on various time scales. Ghanbari et al., from a study of autism spectrum disorder (ASD), reported significant differences in spectral MSEs that were not found in the broadband MSE (19). Furthermore, some reports of the literature suggest that differences in frequency, amplitude, and phase are related respectively to the type of neuronal population, the extent of task involvement, and the excitability of neurons (20–23). For example, in MEG studies, inter-regional neural communication in the human brain has been characterized with both phase–phase synchronization and amplitude–amplitude synchronization (24–26). Consequently, we decomposed neural oscillations into frequency, amplitude, and phase and observed these complexities on various time scales.

For the detailed temporal complexity analyses described above, we analyzed the resting state MEG data using ensemble empirical mode decomposition (EEMD), Hilbert spectral analysis (HSA), and MSE. The idea of combining EEMD and HSA is based on the Hilbert–Huang transform, which combines empirical mode decomposition (EMD) and HSA (27, 28). The EMD method is a frequency decomposition method that is regarded as suitable for processing nonlinear and nonstationary data. Although EMD is often used to remove low-frequency artifacts (29, 30), the method is fundamentally an adaptive time-frequency analysis; it can decompose a time series into some intrinsic mode functions (IMFs) (27, 28, 31–33). An improved version of the original EMD method, EEMD, solves the mode-mixing problem by adding noise (34). Ghanbari et al. (19) described multiple peaks of spectral MSEs decomposed by a bandpass filter (BPF), which might be attributable to the loss of nonlinearity driven by linear frequency decomposition method. To investigate the spectral MSE profiles with intact nonlinearity, we applied the EEMD method instead of other frequency decomposition methods. The spectral time series obtained by EEMD is decomposed further into the amplitude and phase by HSA. In addition, to eliminate the discontinuities in the phase time series, the cosine of the phase is used in the calculation of the phase complexity. Finally, the spectral, amplitude, and phase complexities are evaluated at different time scales using MSE.

This study was conducted to test the usefulness of amplitude complexity and phase complexity for diagnosing AD. Although earlier reports have described that observing complexity on various time scales is useful for the diagnosis of AD, the appropriate interpretation of the time scales remains unclear. For example, some earlier studies have examined different aspects of entropy such as maximum entropy and its time scale (19, 35) and the slope of MSE profile (13, 36). Others have claimed diagnostic significance differing across entropies of various time scales (15, 16). For this study, we prepared a feature pool using an unsupervised neural network (NN) to avoid loss of information that is useful but difficult to interpret. We therefore selected useful features for diagnosis from the obtained features, and diagnosed AD using a support vector machine (SVM). In many clinical studies such as this one, the small sample size poses difficulties that hinder machine learning (ML), especially NN. For this study, we first specifically examined the similarity of the MSE profiles in various brain regions. We then augmented the number of samples used for ML by regarding each estimated region as an individual sample. However, because the MSE profiles in all brain regions do not classify healthy controls (HC) and AD similarly, we first summarized the MSE profiles using a sparse autoencoder (SAE), which is an unsupervised ML method. As reported from an earlier study (37), we used multiple SAEs because they provide a good feature pool. The multiple SAEs learned the MSE profiles in all brain regions to summarize the distribution of sample entropy (SampEn) properly at each time scale. In addition, because these features were not obtained by supervised training, useful features for diagnoses were selected from these features statistically using Fisher’s score, which is a common feature selection (FS) method using linear discriminant analysis. Using features obtained in this manner, HCs and AD patients were classified using SVM. Its usefulness for the diagnosis was evaluated by five-fold double cross-validation (CV). Furthermore, various parameters in the ML architecture were optimized using Optuna, a hyperparameter search software using the Bayesian optimization algorithm (38). Finally, we compared the diagnostic accuracy of this ML architecture (SAE + FS + SVM) with the common supervised ML methods: simple SVM and the FS + SVM.

In summary, our aims were threefold: 1) decomposing neuronal complexity to examine which component is correlated with the aberrant complexity; 2) examining characteristics of the decomposed complexity in AD patients and their relevance to the cognitive function; and 3) examining the utility of ML to improve the diagnostic accuracy further. To this end, we calculated the spectral, amplitude, and phase MSEs using the EEMD, HSA, and MSE method. Then we diagnosed these MSE profiles using SAE, FS, and SVM.

## Methods

### Subjects

The clinical group consisted of 17 subjects (10 men, 7 women), aged 71.7 ± 6.5 years (range 60–80), who were recruited from Kanazawa University Hospital (**Table 1**). The patients fulfilled the National Institute of Neurological and Communicative Diseases and Stroke/Alzheimer’s disease and Related Disorders Association (NINCDS-ADRDA) work group criteria for probable AD (39). Neurological, serological, and magnetic resonance imaging (MRI) tests were performed on these patients to eliminate any other medical condition that might cause dementia. No patient was receiving medications acting upon the central nervous system except donepezil hydrochloride (Dz). Ten patients had taken Dz. The patients were assessed using the Japanese version of the Mini-Mental State Examination (MMSE) (40) and the Wechsler Memory Scale-Revised (WMS-R) (41). The MMSE scores of the clinical group were 22.2 ± 3.7 (range 14–28). The healthy control (HC) group consisted of 21 elderly subjects (13 men, 8 women) aged 68.1 ± 7.3 years (range 55–78); their MMSE scores were 28.7 ± 1.0 (**Table 1**). They had no subjective cognitive impairment. Their WMS-R subscores were not below 1.5 standard deviation of normal range. No HC subject had any personal or family history of psychiatric or neurological disease. All were functioning normally and independently in their daily lives. None was taking central nervous-system-affecting medications. All subjects were right-handed. The Ethics Committee of Kanazawa University Hospital approved the methods. All procedures were performed in accordance with the Declaration of Helsinki. All subjects agreed to participate in the study with full knowledge of the experimental characteristics of the research. After a complete explanation of the study, written informed consent was obtained from each subject.

**Table 1:** Data of the healthy controls (HC) and Alzheimer’s disease (AD) participants.

		HC (*N* = 21)	AD patients (*N* = 17)		Statistical analysis (HC *vs.* AD)	
				Dz (*N* = 10)	T or χ	*P*
Age (years)		68.1 ± 7.3	71.7 ± 6.5	71.3 ± 6.7	1.57	0.125
Gender, (% male)		13 (61.9)	10 (58.8)	5 (50.0)	0.04	0.847
Education (years)		12.3 ± 3.1	11.6 ± 2.6	11.4 ± 2.4	−0.74	0.466
MMSE		28.7 ± 1.0	22.2 ± 3.7	22.2 ± 4.5	−7.77	<0.001
WMS-R	General	102 ± 11	65 ± 13	64 ± 13	9.44	<0.001
	Concentration	105 ± 12	87 ± 17	87 ± 17	3.85	<0.001
	Verbal	101 ± 11	69 ± 12	66 ± 12	8.60	<0.001
	Visual	102 ± 12	67 ± 15	68 ± 16	8.13	<0.001
	Delayed recall	99 ± 12	60 ± 12	61 ± 14	9.85	<0.001
Duration of illness (years)			1.7 ± 1.1	1.7 ± 1.2		

### Measurements

Magnetic fields were measured using a whole-head MEG system for adults at the Laboratory of Yokogawa Electric Corp. in Japan. This system (MEGvision PQA160C; Yokogawa Electric Corp., Japan) included monitoring of 160 channels. The magnetic fields were sampled at 10,000 Hz per channel (bandpass 0.16–2,000 Hz). Resting-state MEG data were recorded for 120 s for each subject with eyes open. In addition, T1-weighted MRI images were acquired (Sigma Excite HD 1.5 T; GE Yokogawa). All subjects had pointed spherical lipid markers placed at the five MEG fiduciary points to enable superposition of the MEG coordinate system onto the MRI. The MRI consisted of 166 sequential slices of 1.2 mm, with a resolution of 512 × 512 points in a field of view of 261 × 261 mm. Individual cortex envelopes were extracted using software for cortical surface-based analysis (15,000 voxels, FreeSurfer 5.1) (42, 43).

### Analyses of Physiological Functions

**Figure 1:**
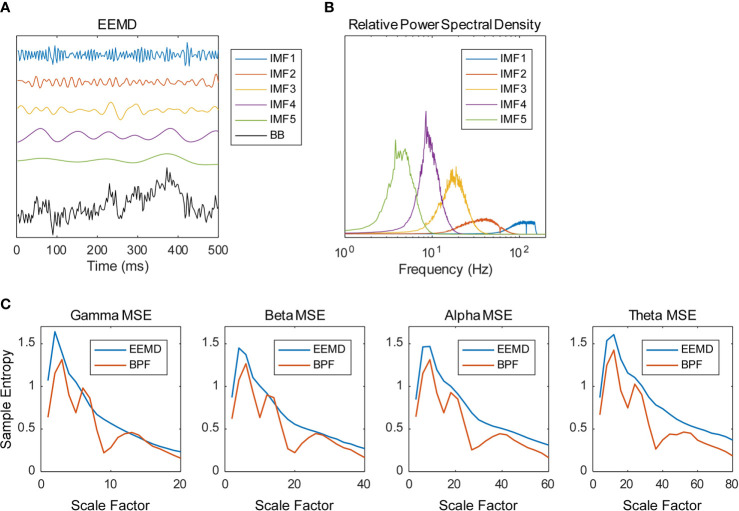
Frequency decomposition by ensemble empirical mode decomposition (EEMD). **(A)** Example of the source time series calculated from magnetoencephalography (MEG) data decomposed into five intrinsic mode functions (IMFs) using the EEMD (BB, broadband). **(B)** Example of the relative power spectral densities of the decomposed time series. **(C)** Multiscale entropy (MSE) profiles obtained using EEMD and bandpass filter (BPF). Source time series were decomposed into four frequency bands using EEMD (IMF 2–5) and BPF (gamma, 33–55 Hz; beta, 16–27 Hz; alpha, 8–14 Hz; theta, 4–7 Hz). Examples of spectral MSE of a healthy control (HC) subject averaged within the temporal regions are shown (gamma—1, 2, 3, …, 20 SF; beta—2, 4, 6, …, 40 SF; alpha—4, 8, 12, …, 80 SF; theta—8, 16, 24, …, 160 SF). The BPF-based spectral MSEs showed multiple peaks, whereas the EEMD-based spectral MSE showed a single peak.

Data analyses of the MEG data presented in this section were performed using software (MATLAB; the MathWorks Inc., Natick, MA and Brainstorm (44); http://neuroimage.usc.edu/brainstorm). The magnetic field data were resampled at 400 Hz with 150 Hz low-pass and 60 and 120 Hz notch filters, segmented for 10 s (artifact-containing segments were excluded) and cleaned using the Signal-Space Projection (SSP) algorithm, removing signals corresponding to blinks and heartbeats. The magnetic time series were transformed into source time series of 148 regions of the Destrieux brain atlas (prefrontal, 16 regions; frontal, 20 regions; central, 18 regions; parietal, 16 regions; occipital, 22 regions; temporal, 42 regions; limbic, 14 regions) (45, 46) using a weighted minimum norm estimation (wMNE) algorithm (47–49) and the scout function of Brainstorm. The source time series were decomposed into five IMFs using EEMD and were then averaged across all 10-s segments. We implemented the EEMD by adding white noise at amplitude of 0.2 standard deviations of the original source time series and calculated an average of 200 ensembles as the IMF (**Figure 1**). **Figure 1B** portrays the relative power spectral densities of the decomposed time series. As an adaptive method, EEMD differs from many other frequency decomposition methods. Although its nature is an advantage of EEMD, the frequency of IMFs varies according to the sampling rate and low pass filtering (LPF) of the time series. Given the conditions of this study (400 Hz sampling frequency; 150 Hz LPF), the peak frequencies of IMFs 1–5 are approximately >100 Hz, 40 Hz, 20 Hz, 10 Hz, and 4 Hz, so we analyzed IMFs 2–5 for the remainder of the analyses and respectively called them the gamma, beta, alpha, and theta bands. The time series were further decomposed into amplitude and phase time series using the HSA and processed using the MSE method. Actually, the MSE method is a temporal complexity analysis method of measuring SampEn on various time scales. Considering a time series = {*x_1_*, *x_2_*,…,*x_N_*}, SampEn can be computed as (15, 36, 50)

$$\text{SampEn}\left(r,m,N\right)=-\ln\frac{A_{m}\left(r\right)}{B_{m}\left(r\right)},$$

where *A_m_(r)* = {number of pairs (*i*,*j*) with $\left|x^{m+1}(i)-x^{m+1}(j)|<r\times SD,i=1,\ldots ,N-m(i\neq j)\right]$/{number of all probable pairs}, and *B_m_(r)* = {number of pairs (*i*,*j*) with $\left|x^{m}(i)-x^{m}(j)|<r\times SD,i=1,\ldots ,N-m(i\neq j)\right]$/{number of all probable pairs}. Therein, $x^{m}\left(i\right)$ is a vector of length *m*, such that $x^{m}\left(i\right)=\left\{x_{i},x_{i+1},x_{i+2},\ldots ,x_{i+m-1}\right\}$, *r* represents the tolerance for accepting matches, *SD* denotes the standard deviation of the time series, and $\left|x^{m}\left(i\right)-x^{m}\left(j\right)\right|$ stands for the Chebyshev distance between $x^{m}\left(i\right)$ and $x^{m}\left(j\right)$. We respectively defined the MSE of each IMF, that of its amplitude, and that of the cosine of its phase, as the spectral MSE, the amplitude MSE, and the phase MSE. We used *m* = 2 and *r* = 0.2 to calculate the MSE values (15). Considering the frequency of each IMF, 20 appropriate scale factors (SFs) were determined (gamma—1, 2, 3, …, 20 SF; beta—2, 4, 6, …, 40 SF; alpha—4, 8, 12, …, 80 SF; theta—8, 16, 24, …, 160 SF). As discussed in *Introduction*, the multiple peaks of spectral MSE reported by Ghanbari et al. (19) were assumed to be attributable to the BPF-derived loss of linearity. As expected, the BPF-based spectral MSEs showed multiple peaks, whereas the EEMD-based spectral MSEs showed a single peak (**Figure 1C**).

For analysis of covariance (ANCOVA) (**Table 2**), the MSE profiles were averaged into seven brain regions (i.e., prefrontal, frontal, central, parietal, occipital, temporal, and limbic). When significant group difference was identified, *post hoc* analysis (**Figure 3**) and correlation analysis with cognitive function (**Table 3** and **Figure 5**) were applied. At the same time, correlation analyses conducted among spectral, amplitude, and phase MSE (**Figure 4** and **Supplementary Figure 1**) and ML (**Table 4** and **Figure 6**) dealt separately with the 148 regions.

**Table 2:** *P* values in ANCOVA for multiscale entropy (MSE) analyses between groups [healthy controls (HC) *vs.* Alzheimer’s disease (AD)] (Bonferroni-corrected for multiple comparisons across all seven regions).

	Broadband		Gamma		Beta		Alpha		Theta	
	Group	Group × SF	Group	Group × SF	Group	Group × SF	Group	Group × SF	Group	Group × SF
Prefrontal	1.0	0.132	1.0	1.0	1.0	1.0	1.0	0.319	1.0	1.0
Frontal	1.0	0.123	1.0	1.0	0.137	1.0	1.0	0.165	1.0	1.0
Central	1.0	0.061	1.0	1.0	0.078	1.0	1.0	0.127	1.0	1.0
Parietal	1.0	0.061	1.0	1.0	0.440	1.0	1.0	0.109	1.0	1.0
Occipital	1.0	0.141	1.0	1.0	1.0	1.0	1.0	0.258	1.0	1.0
Temporal	1.0	0.065	1.0	1.0	0.519	1.0	1.0	**0.039**	1.0	1.0
Lingual	1.0	0.081	1.0	1.0	0.456	1.0	1.0	0.200	1.0	1.0

In bold, P < 0.05.

**Table 3:** Correlation between age, cognitive function, and alpha-band multiscale entropy (MSE) in the temporal region (not corrected for multiple comparison).

		Age	MMSE	WMS-R			
				Concentration	Verbal	Visual	Delayed recall
Spectral—short	R	−0.27	0.09	−0.13	0.25	0.20	0.14
	P	0.099	0.569	0.443	0.137	0.222	0.401
Spectral—long	R	0.18	−0.30	−0.15	−0.12	−0.28	−0.24
	P	0.292	0.066	0.378	0.485	0.093	0.141
Amplitude—short	R	−**0.36**	0.31	−0.03	0.29	**0.36**	0.29
	P	**0.028**	0.056	0.843	0.082	**0.025**	0.080
Phase—long	R	0.25	−**0.42**	−0.28	−0.24	−**0.41**	−**0.36**
	P	0.133	**0.009**	0.092	0.141	**0.010**	**0.025**

R, Pearson’s correlation coefficient; P, P value of the correlation.In bold, P < 0.05.

**Table 4:** Classification results for healthy controls (HC) and Alzheimer’s disease (AD) by support vector machine (SVM), feature selection (FS) + SVM, and sparse autoencoder (SAE) + FS + SVM.

	SVM	FS + SVM	SAE + FS + SVM
Accuracy	0.65 ± 0.05	0.63 ± 0.05	0.69 ± 0.05
Sensitivity	0.72 ± 0.08	0.68 ± 0.09	0.77 ± 0.08
Specificity	0.59 ± 0.09	0.59 ± 0.08	0.62 ± 0.08
AUC	0.70 ± 0.05	0.68 ± 0.05	0.77 ± 0.06

### Machine Learning Methods

**Figure 2:**
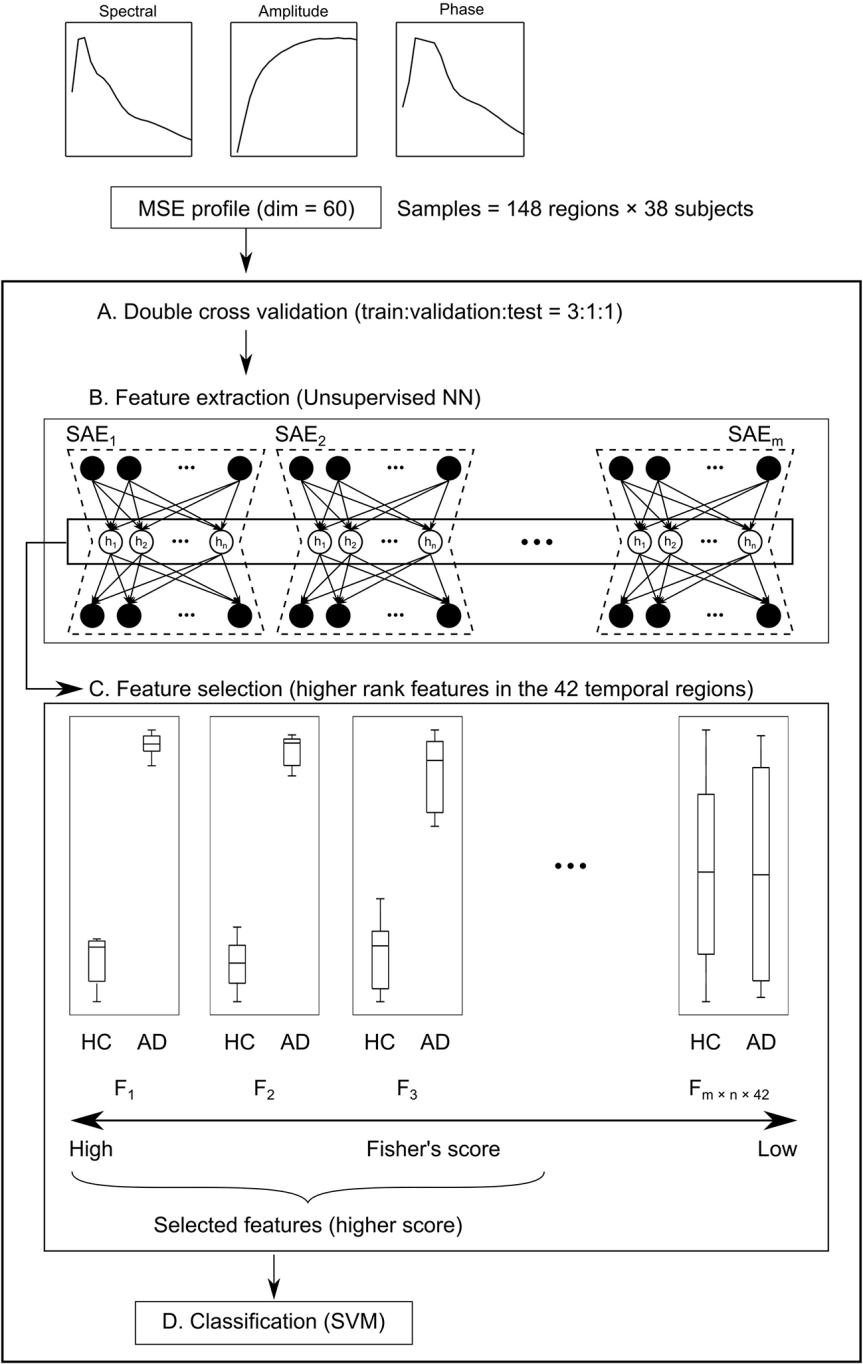
Schema of the machine learning (ML) architecture. **(A)** First, subjects are divided into five groups. Their data are assigned at a train:validation:test ratio of 3:1:1. **(B)** Next, the 60-dim multiscale entropy (MSE) profiles are processed by multiple sparse autoencoders (SAEs) to obtain a feature pool (F_1_ – F_m × n × 148_) using regions × subjects as the number of samples (feature extraction). **(C)** Features of higher Fisher’s score of the temporal regions are selected (feature selection). **(D)** Finally, healthy controls (HC) and Alzheimer’s disease (AD) are classified by the support vector machine (SVM).

The data obtained using complexity analyses have 60 dimensions for each brain region (20 time scales for each of the spectral, amplitude, and phase MSEs). In other words, data from 60-dim MSE profiles × 148 brain regions × 38 subjects were obtained. To examine the diagnostic utility of these multidimensional data from the neuronal complexities, we extracted features by unsupervised NN, feature selection, and SVM (SAE + FS + SVM). First, subjects were divided into five groups by matching the numbers of HC and AD. Then, data from three groups were assigned to the training set, data from one group to the validation set, and data from one group to the test set (**Figure 2A**). The unsupervised training was performed with autoencoders using the training and validation datasets (**Figure 2B**). The autoencoders were sparsified by L1 regularization. Then multiple SAEs were adopted related to Guo et al. (37). The multiple SAEs learned the MSE profiles using the MSE of all the brain regions to summarize the distribution of the SampEn at each time scale properly. In addition, Fisher’s discriminant analysis, a common linear discriminant analysis method, was applied for each feature (**Figure 2C**). Higher rank features were selected. Because the regions were not counted as a sample for the SVM, the number of samples was reduced. Therefore, linear SVM was used to prevent overfitting (**Figure 2D**). Along with this ML architecture (SAE + FS + SVM), two other common ML methods of supervised learning, simple SVM and FS + SVM, were applied to compare the diagnostic accuracy.

In preliminary experiments, the number of SAEs and the number of hidden nodes were tested. Then we adopted 300 SAEs and 70 hidden nodes. Additionally, the number of features selected by the Fisher’s score (100–10,000 features) and the L2 penalty function (1.0 × 10^−3^−1.0) in the SVM were optimized by Optuna for each CV (38). The diagnostic accuracy of this architecture (SAE + FS + SVM) was compared statistically with that of the simple SVM and SVM with Fisher’s score-based feature selection (FS + SVM). In both cases, only the temporal region data were used. In addition, to verify the diagnostic utility accurately, the five-fold double CV was repeated 300 times by changing the grouping of the subjects. SAE and SVM were implemented respectively with the Keras and scikit-learn packages in Python.

### Statistical Analyses

Statistical analyses were conducted using MATLAB and Stata software (Stata Corp., Texas). For demographic and cognitive variables, Student’s *t*-test was used to compare continuous variables. Chi-square testing was used to compare categorical variables. For the spectral MSE, repeated-measures ANCOVA with the groups (AD *vs.* HC), Dz use and sex as the between-subject factor and SF (20 SFs) as within-subject factors were used to test for differences in the SampEn for each region and each frequency band. Bonferroni correction was applied for the regions. The age was treated as covariates. Greenhouse–Geisser adjustment was applied to the degrees of freedom. *Post hoc* independent *t*-tests were used to compare group differences separately for each SF and each decomposed MSE (spectral, amplitude, and phase MSEs). We applied the Benjamini–Hochberg false discovery rate (FDR) for group comparisons to control for multiple comparisons (*q* < 0.1). Additionally, we examined Pearson’s correlations among spectral, amplitude, and phase MSEs, and among the cognitive performance (MMSE score and WMS-R subscores), age, and the SampEn in the temporal region (not corrected for the multiple comparison). We evaluated the diagnostic performance by the area under the receiver operating characteristic (ROC) curve (AUC).

## Results

### Spectral, Amplitude, and Phase Multiscale Entropies

**Figure 3:**
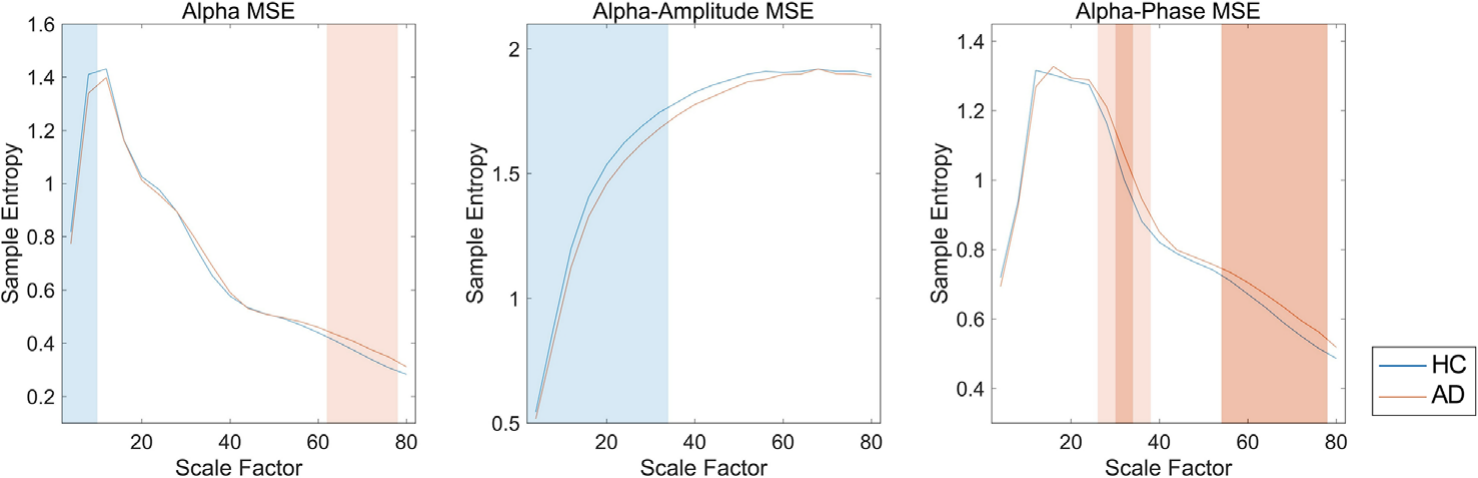
Spectral, amplitude, and phase multiscale entropies (MSEs) of the alpha band in the temporal region. Blue and red lines respectively show healthy controls (HC) and Alzheimer’s disease (AD); blue and red shaded areas respectively show HC > AD and HC < AD; dark and light shaded areas respectively represent *p* < 0.01 and *p* < 0.05. False discovery rate (FDR) *q* corrections were controlled for 20 scale factors (SFs).

We first investigated the broadband and spectral MSE and performed ANCOVA. As a result, a significant group-by-SF interaction was found only for the alpha band in the temporal region (**Table 2**). However, no significant interaction or main effect was observed for Dz and sex. *Post hoc* analysis indicated that the alpha MSE was decreased significantly on short time scales and increased significantly on long time scales in AD patients (**Figure 3**). Furthermore, when the complexity analyses were performed by dividing the amplitude and phase using the HSA, results showed that the amplitude complexity decreased on short time scales and the phase complexity increased on long time scales in AD patients, similar to alpha MSE (**Figure 3**).

**Figure 4:**
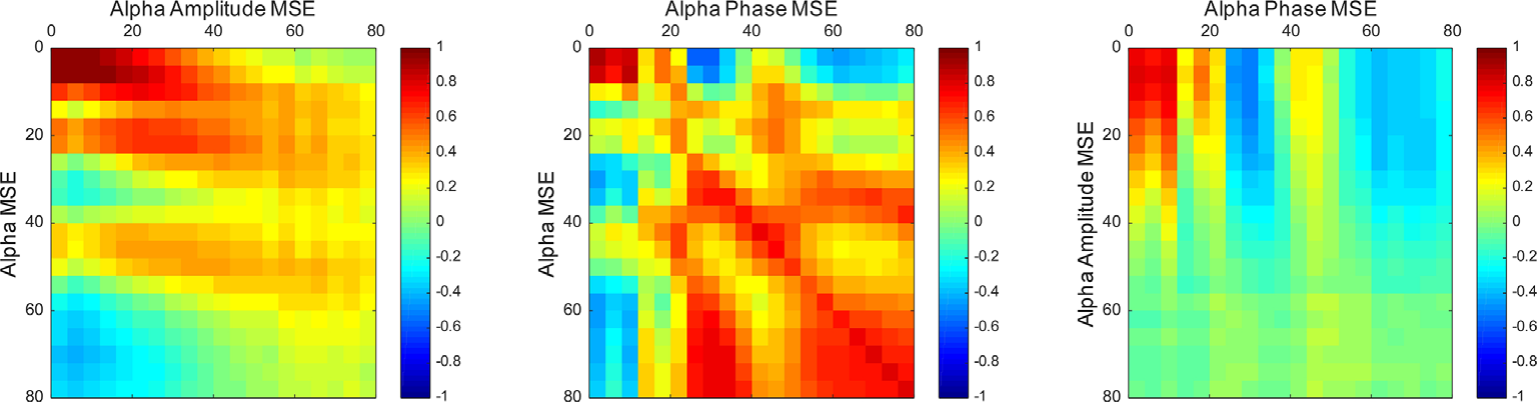
Correlation maps between the spectral, amplitude, and phase SampEn of the alpha band in the temporal region. The spectral complexities were positively correlated with the amplitude complexities on short time scales and the phase complexities on long time scales. Vertical and horizontal axes show scale factors (SFs).

Furthermore, we examined correlations among the spectral, amplitude, and phase SampEn of the alpha band in the temporal region (**Figure 4**). On the ultrashort time scales (SF ≤ 12), the spectral, amplitude, and phase complexities were all positively correlated. The spectral complexities were positively correlated with the amplitude complexities on short time scales and with the phase complexities on long time scales. In addition, the amplitude and phase complexities were inversely correlated in some SFs, but were almost completely uncorrelated in the others. It is particularly interesting that these trends were the same when the regions were extended to the whole brain, but differed at different frequencies. Particularly, the amplitude and phase complexities were uncorrelated at low frequencies, but were inversely correlated at high frequencies (**Supplementary Figure 1**).

### Relation Between Multiscale Entropy Values and Cognitive Function

**Figure 5:**
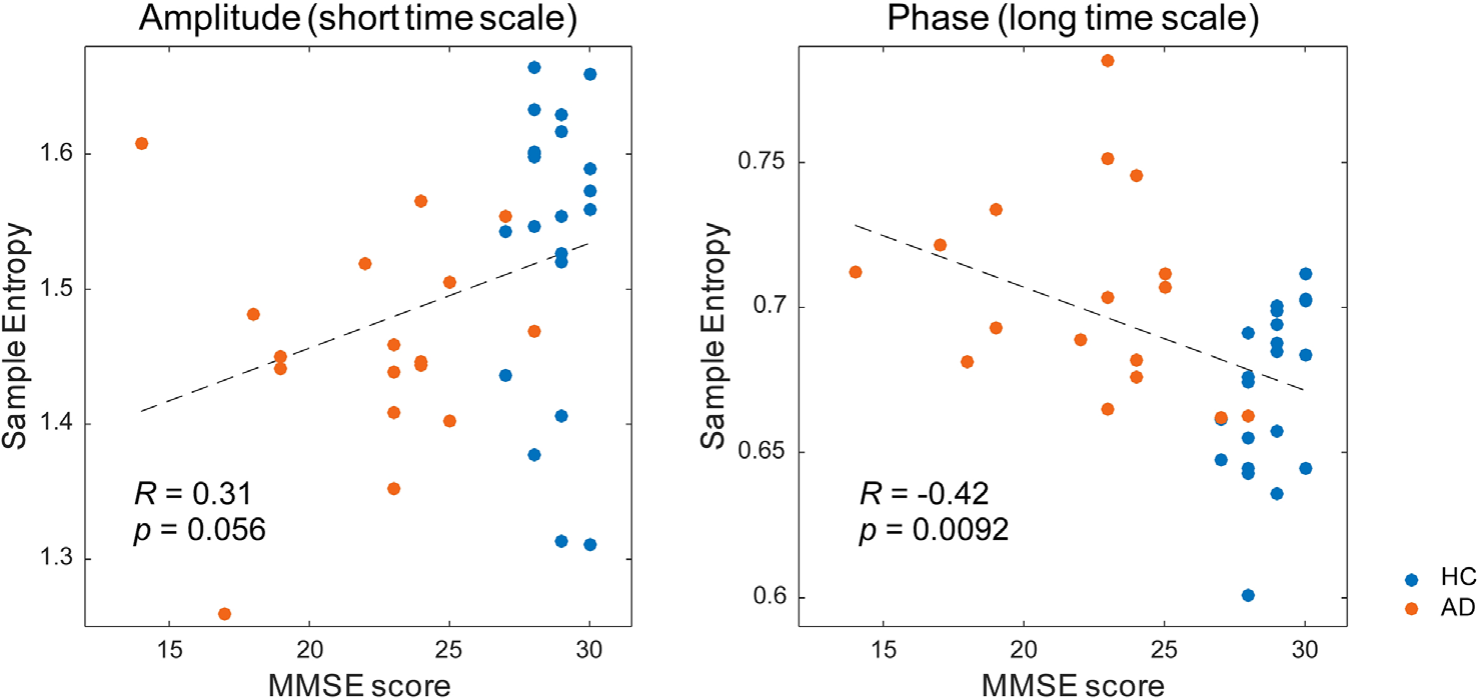
Correlation between the multiscale entropy (MSE) values and the Mini-Mental State Examination (MMSE) scores. The MMSE scores showed a mild positive correlation with the amplitude complexity on the short time scale (SF = 20, *r* = 0.31, *p* = 0.056) and a significant negative correlation with the phase complexity on the long time scale (SF = 60, *r* = −0.42, *p* = 0.0092).

Then, we investigated the relation between the MSE values and cognitive function (**Table 3** and **Figure 5**). The alpha spectral complexity was found to have no significant correlation with age or cognitive decline on either a short or long time scale. The amplitude complexity on the short time scale (SF = 20) showed significant positive and negative correlation, respectively, with visual memory subscores on the WMS-R and the age of the subjects. Furthermore, the phase complexity on the long time scale (SF = 60) was found to have significant negative correlation with MMSE and visual memory and with delayed recall subscores in the WMS-R (not corrected for multiple comparisons).

### Diagnostic Performance in Detecting Alzheimer’s Disease Using Machine Learning Methods

**Figure 6:**
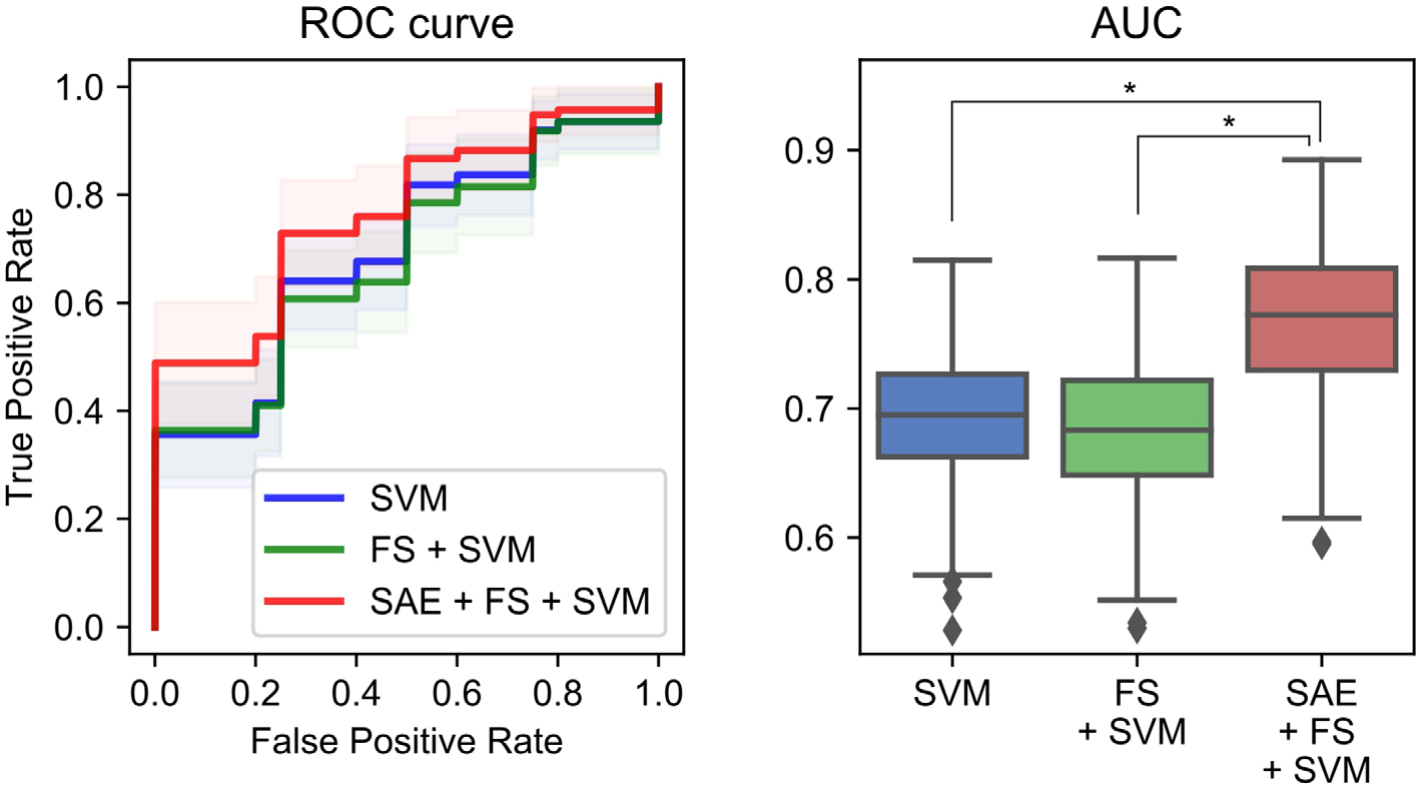
Performance of a combination of the complexity characteristics and machine learning (ML) methods for the diagnosis of Alzheimer’s disease (AD). The area under the receiver operating characteristic curve (AUC) was 0.70 ± 0.05 for the simple support vector machine (SVM), 0.68 ± 0.05 for the feature selection (FS) + SVM, and 0.77 ± 0.06 for the sparse autoencoder (SAE) + FS + SVM. *p < 0.001.

Additionally, we used ML methods to test the diagnostic utility of these complexities (see *Machine Learning Methods* for details). We specifically examined the similarity of the MSE profiles in various brain regions. First, we augmented the number of samples by regarding each region as an individual sample. Also, we performed unsupervised training with multiple SAEs (**Figure 2B**). The higher rank features in the temporal regions were selected by Fisher’s score (**Figure 2C**). The final classification of HC and AD was performed using linear SVM (**Figure 2D**). Results show that our proposed SAE + FS + SVM method performed better than either the simple SVM or the FS + SVM (**Figure 6** and **Table 4**).

## Discussion

Earlier studies have explored the hypothesis that not only the frequency but also the amplitude and phase respectively correspond to individual functions. Many clinical and experimental studies have specifically examined this theory (20–23). Therefore, the key concept of this study is the decomposition of neural oscillation and their influence to the temporal complexity results in AD patients. Practically, MSE analysis captures a wide range of physiological systems. It has been applied fruitfully in clinical settings. However, far fewer studies have applied decomposition method to MSE analysis. For this study, the MEG signal was decomposed using the combination of the EEMD and HSA; it was then applied to MSE analysis. Furthermore, we tested the usefulness of these decomposed complexities in diagnosing AD using ML methods.

### Group Difference (AD *vs.* HC) and Relevance to Cognitive Function

In this study, the AD patients showed lower amplitude complexity and higher phase complexity in the alpha band in the temporal region than the HC subjects did (**Table 2** and **Figure 3**). The temporal region has been implicated as the primary site of dysfunction in AD (4, 5, 51). Also, M/EEG studies have shown spectral changes in the temporal and parietal regions in AD and MCI patients (10, 12). In addition, broadband MSE alterations have been observed in various brain regions, mainly in the temporal and parietal regions in AD patients (13, 15, 16). In the present study, significant differences were found only in the temporal region (**Table 2**), probably because neuronal disconnection starts in the temporal region in AD patients. However, it remains unclear why only the temporal region was identified in this study, unlike earlier studies. Two explanations can be considered as reasons. First, we compared the mild patient group with HC (see *Limitations*). The temporal regions are altered in the early stages of AD. Many earlier studies have suggested that structural and functional alterations of temporal region are useful for diagnosis (11, 12, 52, 53). Second, we were able to reduce the volume conduction by performing the source localization method, unlike EEG, which led to better spatial resolution, although we cannot rule out the possibility that it is simply attributable to the lower sensitivity. In any case, the finding of localized alteration in the temporal region in mild AD is consistent with findings presented in earlier reports indicating the temporal region as the primary site of dysfunction in AD. Furthermore, using the spectral MSE, we were able to identify alterations only in the alpha band complexity. Earlier reports described that alpha oscillation is associated with memory function (54, 55). Recent reports of some studies have described that enhancement of alpha oscillation by neuromodulation improves memory performance (56–58). The altered alpha band complexity observed in the present study might reflect disturbance of the memory function in AD.

It is noteworthy that the complexity of amplitude and phase demonstrated opposite findings across short and long time scales (**Figure 3**). Similarly to our study, earlier studies have found reversal relations between short and long time scales in broadband MSE in AD (13–16) and coma patients (59). Those relations were inferred as dependent on the frequency (15, 35, 60). However, in this study, the reversal relation, which is dependent on the time scale, was observed even after frequency decomposition (**Figure 3**). Additionally, we found that spectral complexity on short time scales correlates with amplitude complexity and that spectral complexity on long time scales correlates with phase complexity (**Figure 4**). Therefore, the time scale of the complexity depends not only on the frequency but also on the components of the amplitude and phase. Regarding cognitive function, the amplitude and phase complexity were found to be correlated significantly with cognitive function in a different manner (**Table 3** and **Figure 5**). Specifically, the amplitude complexity was found to be correlated with the age and visual memory subscore in the WMS-R, whereas the phase complexity correlated with MMSE, visual memory, and delayed recall subscores. These results suggest a differential role of amplitude and phase complexity in the neural basis of cognitive functions in AD. Because the multiscale temporal complexity of neural oscillations is assumed to reflect the influences from the past neural processes through feedback loops at multiple hierarchical levels of cortical processing (15, 61), we infer that the altered complexity of phase and amplitude in AD are generated separately by the disconnection of several feedback loops. It is particularly interesting that Courtiol et al. (35) reported that older adults selectively showed lower EEG complexity than younger adults on short time scales. Herein, we speculate that reduced amplitude complexity with aging on short time scales might be related to age-related memory dysfunction, whereas elevated phase complexity might reflect AD-derived cognitive decline. Results of another study have also suggested that the decline in visual memory function might be attributable not only to memory function, but also to impaired visual processing (62). Considering the fact that no correlation of spectral MSE with cognitive function was identified, decomposing the spectral MSE into amplitude and phase might yield information in addition to that already identified using conventional spectral MSE.

### Improvement of Diagnostic Performance Using Unsupervised Machine Learning

We have assessed a method of interpreting multidimensional information about these complexity characteristics. For this study, which was designed for AD diagnosis, the proposed method improved the diagnostic performance. In general, when diagnostic performance is poor, even though individual feature values are excellent, it can be suspected that 1) information was not well interpreted or 2) information that was unnecessary for diagnosis was included. With respect to 1), the appropriate interpretation of the time scales of MSE remains unclear, as described in *Introduction* (13, 15, 16, 19, 35, 36). For the present study, we tried to solve 1) using NN. Although a large sample size is required for NN, we took advantage of the similarity of MSE profiles across all regions to augmented data by regarding each region as an individual sample. However, we needed to apply unsupervised NN to learn the MSE profile instead of supervised NN to learn the diagnosis, because not all regions classify HC and AD similarly. Using supervised learning without splitting the regions would be ideal to, but we abandoned those benefits to apply NN for feature extraction. Then the output was applied to the supervised classifier (i.e., SVM) to learn the diagnosis as described below. Autoencoders of several kinds were used as the unsupervised feature learning methods in earlier studies, demonstrating their utility (63–65). For instance, Guo et al. (37) reported that a good feature pool is obtainable using multiple SAEs for the diagnosis of ASD. However, because unsupervised training does not learn “diagnosis,” many unnecessary features are expected to be extracted. Therefore, we evaluated the discriminating power of each feature using Fisher’s score and chose higher rank features to solve 2) (66, 67). Finally, because nonlinear kernel SVM classifiers often encounter overfitting (68), we used linear kernel SVM for supervised learning at the last part of our ML architecture (**Figure 2**). In preliminary experiments, the performance was better when the number of SAEs was larger. Therefore, 300 SAEs were prepared. In addition, 70 nodes were adopted, because the performance was better when the number of nodes in the hidden layer was 40 or 70. In addition to the proposed method combining an unsupervised nonlinear ML method (SAE + FS + SVM), two more conditions were prepared using only common supervised linear discrimination methods for comparison (simple SVM and FS + SVM). The simple SVM performs neither 1) nor 2); the FS + SVM performs only 2). The condition for only 1) was not performed because the feature dimensions for SVM are too large. As a result of double CV, SAE + FS + SVM improved the diagnostic accuracy of the simple SVM and FS + SVM significantly (**Figure 6**). The AUC was 0.70 ± 0.05 for the simple SVM and 0.68 ± 0.05 for the FS + SVM, which was not very high, probably because of the mild severity of the AD group (see *Limitations*). However, SAE + FS + SVM was found to be associated with significant improvement in AUC (0.77 ± 0.06). This result suggests that the multidimensional information of oscillatory components (i.e., spectral, amplitude, and phase) and time scales was interpreted successfully by the SAE and that unnecessary information was excluded properly by the FS. Consequently, the SVM-based diagnostic accuracy was improved using SAE and FS, in spite of consideration of each region separately and using unsupervised NN. When similarities are found in subgroups such as regions, as in the present study, unsupervised training and feature selection can extract the potential multidimensional information and improve the diagnostic performance. However, ways other than splitting the regions should also be explored to increase the sample size.

### Meanings of Spectral, Amplitude, and Phase Complexity and Their Time Scales

Earlier studies revealed that SampEn on each time scale correlates with the band power of the respective frequencies (15). However, a study by Ghanbari et al. (19) and the present study showed that examining MSE at each frequency band provides meaningful information to diagnose ASD and AD. In other words, neural oscillations have a two-dimensional meaning on the frequency × time scale. Additionally, we identified the possibility that short time scales correspond to amplitude and that long time scales correspond to phase as candidate meanings for the time scales. These relations were consistent across all brain regions (**Supplementary Figure 1**), but the profiles differ by frequency band. This definite simple relation of frequency and time scale might result from the beneficial use of EEMD, which ignores artifacts and which enables achievement of a single peak of the MSE profile. Additionally, amplitude and phase complexities apparently have different physiological meanings (**Table 3** and *Group Difference (AD vs. HC) and Relevance to Cognitive Function*). This observation might support the hypothesis that a narrow band spectral complexity corresponding to an independent neural population is influenced by different neural processes on different time scales. Furthermore, for another study, we compared the decoding accuracy of neural decoding by features of two types, band power and multiscale complexity, for the amplitude and phase of each frequency. Results show that the latter had better decoding accuracy (69). This finding suggests that the MSE might have improved the decoding performance using information about history. The finding also supports the hypothesis that different oscillatory components reflect different neural functions (20–23).

Nevertheless, it remains unclear whether the manner of tradeoff between amplitude and phase complexity is always true, or only true in certain cases. Three clues point to resolution of this point. First, the manner of tradeoff differs for each frequency. At high frequencies, amplitude and phase complexity share a tradeoff relation, but at low frequencies, inverse correlation was found only at a few SFs (**Supplementary Figure 1**). Second, amplitude and phase complexity are correlated to different profiles of the subjects. Amplitude complexity is correlated with the age and visual memory subscore in the WMS-R, whereas phase complexity is correlated with MMSE and visual memory and delayed recall subscores in the WMS-R, but not with age (**Table 3** and **Figure 5**). Third, earlier studies indicated no manner of tradeoff in ASD (19, 70), schizophrenia (71), and aging (35, 36). Nevertheless, most of these studies examined broadband complexity. By contrast, the present study examined decomposed complexity. Regarding these three points, amplitude and phase complexity are considered not always to represent a tradeoff. This finding suggests the importance of separately examining amplitude and phase complexity.

### Limitations

As described in *Improvement of Diagnostic Performance Using Unsupervised Machine Learning*, the diagnostic performance was not very high compared to that found in earlier studies (13, 15, 16), probably because the severity of the AD in patients examined for this study was mild. Considering the relation between MMSE and clinical dementia rating (CDR) in earlier studies (16, 51, 72–74), the patient group in this study can be regarded as CDR = 0.5–1.0, i.e., very mild to mild cases. The MMSE score in the AD group was 22.2 ± 3.7 in this study, but it was 13.1 ± 5.9 as reported by Escudero et al. (13) and 15.9 ± 4.5 as reported by Mizuno et al. (15). The duration of illness was 1.7 ± 1.1 years in this study, but it was reported as 2.7 (0.0–9.2) years by Mizuno et al. (15). Yang et al. (16) divided AD patients by severity and found significant differences in various regions in the moderate to severe group (CDR ≥ 2.0; MMSE, 11.5 ± 4.6; 2.2 ± 2.1 years duration), but no difference was found for the very mild group (CDR = 0.5; MMSE, 24.2 ± 4.2; 1.2 ± 0.9 years duration) and mild group (CDR = 1.0; MMSE, 19.0 ± 5.2; 2.3 ± 2.2 years duration) in the temporal region (16). The present study found significant differences in MSE values, even though the AD group severity was milder than that of the mild group (CDR = 1.0) reported by Yang et al. (16). This result rather suggests that the decomposed complexities are useful features to diagnose mild AD. The diagnostic accuracy of our proposed method was inferior to that of MMSE (accuracy, 0.88 ± 0.04; sensitivity, 0.96 ± 0.03; specificity, 0.81 ± 0.07; and AUC, 0.98 ± 0.01), probably because NINCDS-ADRDA itself includes MMSE as a criterion. Currently, the diagnosis of AD is based largely on clinical symptoms (e.g., cognitive decline), even at the pre-dementia stage. Many of the patients are diagnosed as having AD only when their symptoms have already advanced. In fact, earlier studies have suggested that histological changes such as amyloid plaque and tau protein progress insidiously for decades before the AD diagnosis (1–3). Therefore, potential clinical benefits of objective biomarkers have been studied intensively for diagnosing the early stage of AD. Although the potential utility of complexity analysis for early diagnosis was not warranted, clinical benefits of our approach were demonstrated even in the mild stage of this disease. Some earlier reports have described that local oscillatory changes might be exhibited in the early stage of AD (9, 10, 75). A structural MRI report has described a study that revealed local disconnection in the medial temporal region in MCI and early AD patients (4). Another report described a study showing correlation between the medial temporal atrophy and memory impairment (51). These results suggest that early AD might present only local changes structurally and functionally. Therefore, examining local changes in temporal complexity might be more useful than either connectivity or network analysis for diagnosing early AD. Future studies must investigate the usefulness of complexity analysis for preclinical subjects.

As described in *Group Difference (AD vs. HC) and Relevance to Cognitive Function*, the temporal complexity of neural oscillations is assumed to reflect the influences of the history of multiple hierarchical feedback loops (15, 61). However, feedback loops consist of multiple neural populations. Particularly, the complexity on long time scales is assumed to reflect the influence of long-distance feedback loops. Therefore, we might be able to observe the effects of feedback loops more directly by examining the complexity of multiple time series, such as neural complexity (76, 77). Additional studies examining multidimensional complexity will be necessary to confirm these findings.

Additionally, we used only temporal regions for the classification of HC and AD to examine time scale information of complexity specifically. However, information from other regions might enrich the input and improve classification performance.

## Conclusion

This study evaluated the amplitude and phase complexity of the MEG data in AD patients and examined their relevance to clinical features. Additionally, we tested some ML methods to improve diagnostic performance. The alpha amplitude and phase complexities in the temporal region were found to have significant difference between AD and HC. They showed significant correlation with cognitive function in a different manner. Additionally, correlation among spectral, amplitude, and phase complexity yielded different profiles depending on the frequency bands. Furthermore, we demonstrated the usefulness of SAE + FS + SVM for improving diagnostic performance.

## Data Availability Statement

The datasets generated for this study will not be made publicly available because of the non-disclosure agreement.

## Ethics Statement

The studies involving human participants were reviewed and approved by the Ethics Committee of Kanazawa University Hospital. The patients/participants provided their written informed consent to participate in this study.

## Author Contributions

NF designed the study. NF, YN, SN, and MKa conducted data analyses. TT, AY, YM, and MKi supervised the research. NF wrote the first draft of the manuscript. YN, TT, and TH revised the manuscript. All authors contributed to the article and approved the submitted version.

## Funding

This work was supported by the Center of Innovation Program and CREST (Grant Number JPMJCR17A4) from the Japan Science and Technology Agency (https://www.coistream.osaka-u.ac.jp/en) and a grant from Novartis Pharma. The funder had no role in the study design, data collection and analysis, decision to publish, or preparation of the manuscript.

## Conflict of Interest

The authors declare that the research was conducted in the absence of any other commercial or financial relation that might be construed as a potential conflict of interest.
