# Supplementary material for: Decomposed Temporal Complexity Analysis of Neural Oscillations and Machine Learning Applied to Alzheimer’s Disease Diagnosis
Source: Front Psychiatry. 2020 Sep 3;11:531801. doi: 10.3389/fpsyt.2020.531801 (PMC7495507; doi:10.3389/fpsyt.2020.531801)
Supplement: Supplementary file 1 [file DataSheet_1.docx]

Supplementary Materials

# Supplementary Figures


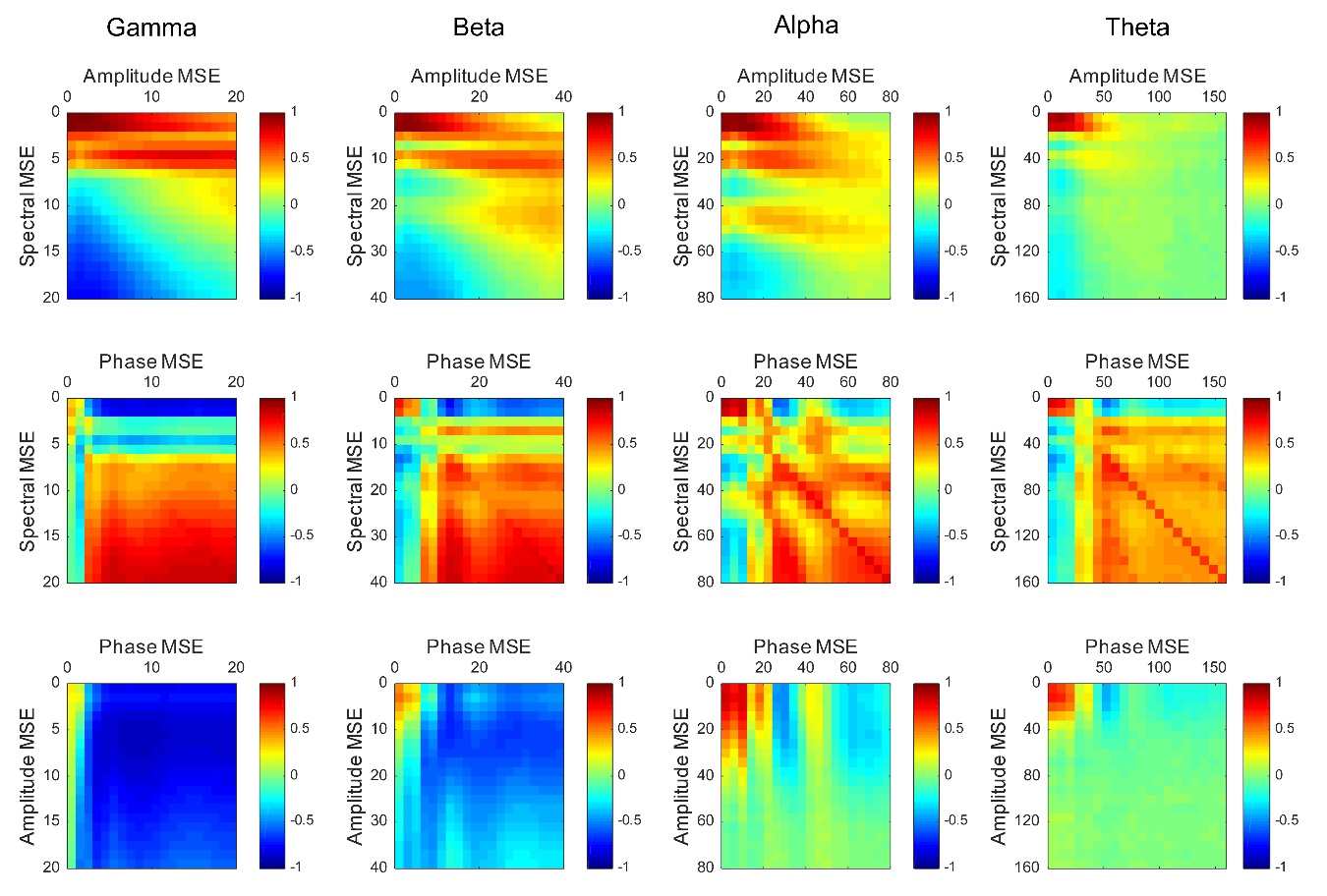


**Supplementary Figure 1.** Correlation maps between the spectral, amplitude, and phase SampEn values for all 148 brain regions. Correlation patterns depend on the frequency. Particularly, the amplitude and phase complexities are uncorrelated at low frequencies, but they are inversely correlated at high frequencies. Alpha band complexities in all brain regions are almost identical to those in the temporal regions. Vertical and horizontal axes show SFs.
